# A retrospective study of first‐line therapy and subsequent pyrotinib treatment in advanced lung adenocarcinoma with HER2 mutations

**DOI:** 10.1002/cam4.7335

**Published:** 2024-06-24

**Authors:** Li Wang, Yueran Wu, Zhixuan Ren, Xiangling Chu, Jianing Chen, Li Liu, Jing Zhao, Xin Yu, Mengqing Xie, Chunxia Su

**Affiliations:** ^1^ Department of Medical Oncology, Shanghai Pulmonary Hospital and Thoracic Cancer Institute Tongji University School of Medicine Shanghai People's Republic of China; ^2^ Department of Chronic Disease Prevention and Control Jiujiang City Center for Disease Control and Prevention Jiangxi China; ^3^ Department of Radiation Oncology Huadong Hospital Affiliated to Fudan University Shanghai People's Republic of China

**Keywords:** efficacy, first‐line treatment, HER2‐mutant, lung adenocarcinoma, pyrotinib

## Abstract

**Objectives:**

HER2 is an infrequently mutated driver gene in non‐small cell lung cancer (NSCLC). At present, there has been no comprehensive large‐scale clinical study to establish the optimal first‐line treatment strategy for advanced lung adenocarcinoma (LUAD) with HER2‐Mutant. Besides that, the effectiveness and safety of pyrotinib, a pan‐HER inhibitor, in the context of NSCLC are still undergoing investigation.

**Materials and Methods:**

In this study, we conducted a retrospective data collection of HER2‐Mutated advanced LUAD who received first‐line treatment and pyrotinib between May 2014 and June 2023. Patients treated with chemotherapy, chemotherapy + immune checkpoint inhibitors (ICIs), chemotherapy + bevacizumab and pyrotinib in first‐line treatment. Furthermore, we collected data on the efficacy and safety of pyrotinib in these patients after disease progression. The main endpoint of the study was progression‐free survival (PFS).

**Results:**

In the final analysis, 89 patients were included in the first‐line cohort and 30 patients were included in the pyrotinib cohort. In the first‐line treatment cohort, chemotherapy + ICIs, chemotherapy + bevacizumab, and pyrotinib exhibited notable survival benefits compared to chemotherapy (median PFS: 9.87 vs. 7.77 vs. 7.10 vs. 5.40 months, *p*‐value < 0.05). Furthermore, patients with a first‐line treatment PFS of less than 6 months may potentially benefit from subsequent treatment with pyrotinib (median PFS: 7.467 vs. 3.000, *p*‐value = 0.0490).

**Conclusions:**

In the first‐line treatment of HER2‐Mutant LUAD, regimens involving combinations like chemotherapy + ICIs, chemotherapy + bevacizumab, and pyrotinib may confer enhanced survival advantages compared to chemotherapy. Nevertheless, no significant distinctions were observed among these three treatment strategies, underscoring the imperative to identify biomarkers for the discerning selection of suitable therapeutic modalities. Moreover, patients with suboptimal response to first‐line treatment may potentially derive more benefit from pyrotinib.

## 1 INTRODUCTION

Nowadays, lung cancer is the second most common cancer and the leading cause of cancer death worldwide among various cancers.^1^ With the rapid development of targeted therapies, various targeted drugs have significantly improved the prognosis of patients harboring driver gene mutations, including epidermal growth factor receptor (EGFR), anaplastic lymphoma kinase (ALK), and c‐ros oncogene 1 receptor tyrosine kinase (ROS‐1).^2^, ^3^, ^4^, ^5^ The human epidermal growth factor receptor 2 (HER2, also known as ERBB2), as one of the rare driver genes in NSCLC, has a mutation rate ranging from 1.4% to 6.7% in Asian populations.^6^, ^7^ The three main forms of HER2 alterations are HER2 gene amplification, HER2 overexpression, and HER2 mutations.^8^ Despite its significance, clinical research on HER2 as a rare driver gene target in advanced NSCLC is still limited.

At present, Immunotherapy has shown convincing clinical benefits, significantly prolonging the survival of advanced NSCLC patients.^9^, ^10^, ^11^, ^12^ Immunotherapy has been recommended as the first‐line treatment for advanced NSCLC patients. Some large randomized clinical trials have found that immunotherapy is less effective in advanced NSCLC patients with driver genes such as EGFR and ALK.^13^, ^14^ However, there is a limited number of prospective studies investigating the negative regulatory impact of the HER2 gene on the immune microenvironment and the effectiveness of immunotherapy in this population. In patients with HER2‐mutant NSCLC treated with first‐line chemotherapy, the ORR was 36% and the PFS of 5.1 months.^15^ Moreover, a retrospective study of first‐line ICI plus chemotherapy reported an ORR of 52% and a median PFS of 6 months.^16^ Numerous studies have demonstrated that post‐chemotherapy prognoses of NSCLC patients with HER2 alterations are unfavorable, with a median PFS of 4.9–5.9 months and median OS of 9.9–10.7 months in first‐line or second‐line treatment.^17^, ^18^, ^19^, ^20^ Based on some retrospective study data, the effectiveness of ICIs combined with chemotherapy in the first‐line treatment of advanced NSCLC patients with HER2 mutations remains controversial.^16^, ^21^ According to the National Comprehensive Cancer Network (NCCN) guidelines for NSCLC, first‐line treatment for HER2‐mutant NSCLC patients is still recommended to follow the approach used for those without driver gene mutations.^22^ The therapeutic value of ICIs in first‐line treatment for HER2‐mutated NSCLC still needs comprehensive evaluation through clinical research.

Pyrotinib, a pan‐HER inhibitor, attracts increasing attention as a potential treatment for HER2‐positive solid tumors. Pyrotinib has been recommended by the National Medical Products Administration (NMPA) in China for treating patients with advanced or metastatic HER2‐positive breast cancer.^23^ In a Phase II trial, the ORR after first‐line pyrotinib treatment was 35.7%, with a median PFS of 7.3 months and a median OS of 14.3 months.^24^ Moreover, according to multiple phase II clinical studies on pyrotinib treating advanced LUAD with HER2 mutations, the observed ORR ranged from 19.2% to 30.0%, with a median PFS of 5.6–6.9 months and a median OS of 10.5–14.4 months.^25^, ^26^ Moreover, there is still a lack of real‐world studies on pyrotinib in HER2‐mutated NSCLC. The efficacy and safety of pyrotinib still lack sufficient evidence in NSCLC, and it has not yet obtained approval for this indication in NSCLC. HER2‐targeted antibody‐drug conjugates (ADCs) have made significant strides in breast cancer and gastric cancer. The DESTINY‐Lung01 and Lung02 studies have confirmed the anti‐tumor activity of Trastuzumab Deruxtecan in patients with HER2‐mutated advanced NSCLC.^27^, ^28^ However, ADCs still lack large‐scale phase III prospective clinical studies to substantiate their efficacy in NSCLC.

In summary, the optimal first‐line treatment strategy and the best combination regimen for HER2‐mutated LUAD patients remain to be elucidated. The effectiveness and safety of pyrotinib in HER2‐mutated LUAD patients also require further investigation. In this study, we conducted a retrospective review of our institution's case series involving HER2‐mutant advanced LUAD patients who underwent first‐line treatment. Moreover, we conducted an efficacy analysis for patients who received pyrotinib in subsequent lines of treatment following disease progression during the first‐line treatment.

## 2 MATERIALS AND METHODS

### 2.1 Study design and population

This single‐center retrospective study was supervised in compliance with the regulations outlined in the Declaration of Helsinki and sanctioned by the Ethics Committee of Shanghai Pulmonary Hospital. In this study, we collected data from all consecutive unresectable stage IIIB/IIIC or IV LUAD patients with HER‐2 Mutant according to the seventh edition of the International Association for the Study of Lung Cancer staging system. These patients received first‐line treatment at the Shanghai Pulmonary Hospital, China, from May 2017 to June 2023. Furthermore, we collected data on the efficacy and safety of pyrotinib in after first‐line cohort treatment progression. The population is primarily divided into the first‐line treatment cohort and the pyrotinib cohort.

In the first‐line treatment cohort, the inclusion criteria were as follows: (1) age ≥ 18 years, (2) HER2 mutations detected at the time of initial diagnosis through next‐generation sequencing (NGS) or ADx HER2 Mutation Detection Kit (Amoy Diagnostics, Xiamen, China) in tumor tissue or liquid biopsy samples, (3) without EGFR sensitizing mutations and ALK rearrangements, and (4) receipt of first‐line treatment. Additionally, patients treated with afatinib, ICI monotherapy, or an angiogenesis inhibitor plus ICI combined with or without chemotherapy, as well as those without available data for efficacy evaluation, were excluded from the efficacy analysis.

In the pyrotinib cohort, the inclusion criteria were as follows: (1) age ≥ 18 years, and (2) receipt of pyrotinib. Besides that, patients treated with pyrotinib combined with other therapies, discontinued for non‐medical problems, as well as those without available data for efficacy evaluation, were excluded from the efficacy analysis.

In two cohorts, PD‐L1 expressions were detected using a Dako 22 C3 pharmDx test kit. The PD‐L1 tumor proportion score (TPS) was calculated as the percentage of ⩾100 viable tumor cells with complete or partial membrane staining. PD‐L1 negative and positive were defined as the PD‐L1 expression ≤0, and PD‐L1 expression ≥1, respectively. The ADx HER2 Mutation Detection Kit (Amoy Diagnostics, Xiamen, China) can detect various HER2 mutation types, including 12‐bp exon 20 insertion, G776 mutations, 9‐bp exon 20 insertion, V777L mutation, and L755p mutation.^25^ All clinical data were extracted from electronic records.

The primary endpoints of this study were PFS and safety. PFS was calculated as the time interval between the initiation of first treatment and disease progression or death for any cause, whichever occurred first. Baseline images of measurable target lesions were obtained with computed tomography of the chest and abdomen and magnetic resonance imaging of the brain. Guidelines from the RECIST version 1.1 were used to classify a complete response (CR), partial response (PR), stable disease (SD), or progressive disease (PD). The severity of adverse events was graded according to the Common Terminology Criteria for Adverse Events (CTCAE) version 5.0.

### 2.2 HER‐2 mutant LUAD from public databases

We further investigated the gene alterations in patients with HER‐2 Mutant LUAD. Firstly, we analyzed the mutation frequency and types of HER2 in the lung cancer population using the cbioportal database. Subsequently, we selected 13 HER2‐mutant patients who underwent immunotherapy and further characterized their mutation profiles, aiming to identify suitable biomarkers for screening patients eligible for immunotherapy.

### 2.3 Statistical analysis

Statistical analyses were conducted using GraphPad Prism 9.0 and R 4.2.2. Chi‐square or Fisher's exact test was used to compare proportions. PFS were assessed using the Kaplan–Meier method. PFS values between different subgroups were compared using the log‐rank test (two‐sided), and the corresponding hazard ratios (HRs) and 95% confidence intervals (CIs) were estimated using the Cox proportional regression model. Cox proportional hazard model was applied for multivariate analysis. *p* < 0.05 was considered statistically significant.

## 3 RESULTS

### 3.1 The first‐line treatment cohort

**FIGURE 1:**
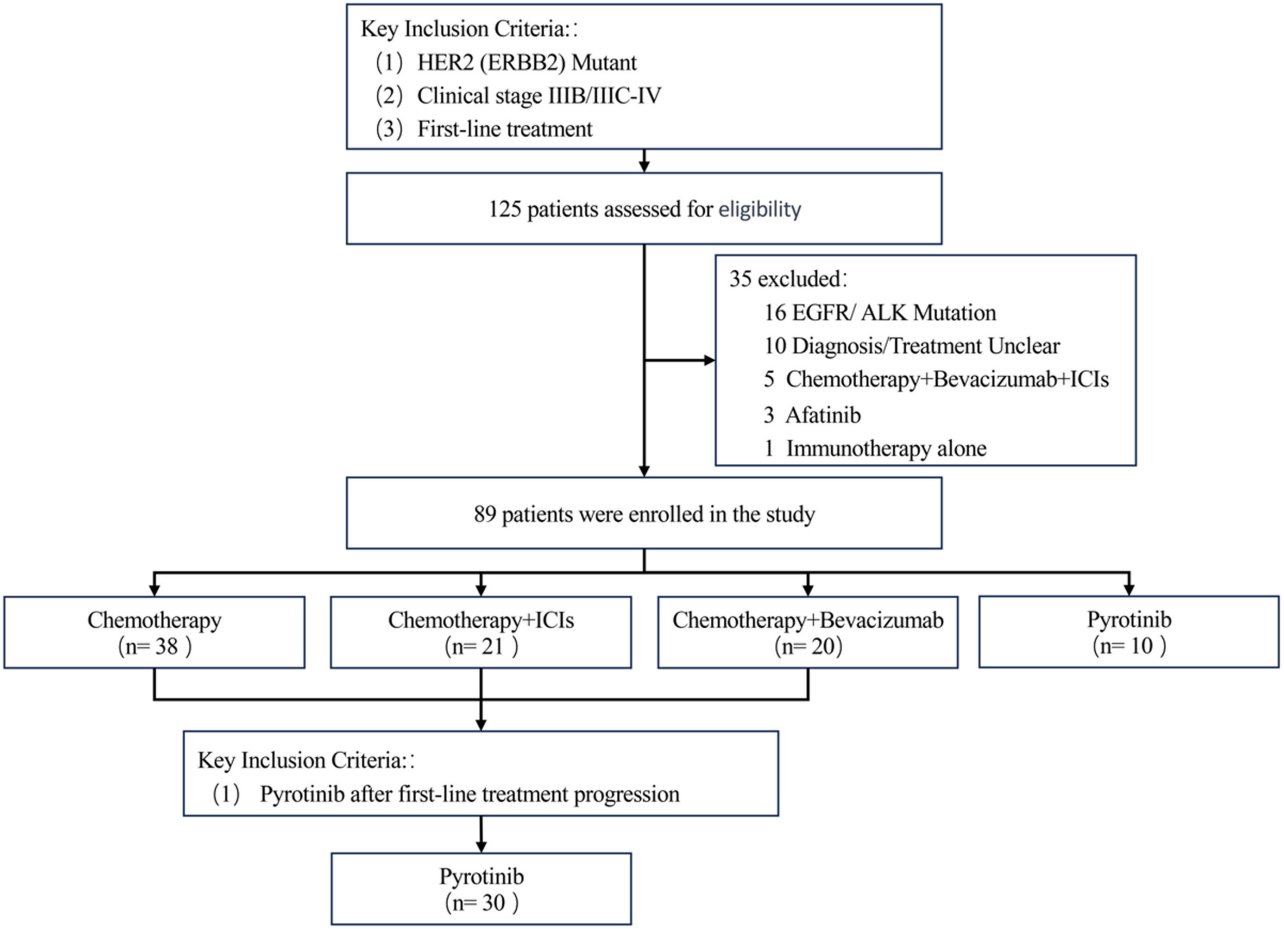
Study workflow.

A total of 89 patients were identified who met the study inclusion criteria (Figure 1). In the first‐line treatment cohort, 38 patients received chemotherapy, 21 received chemotherapy + ICIs, 20 patients received chemotherapy + bevacizumab, and 10 patients received pyrotinib. Detailed patient and treatment characteristics can be found in Table 1. Apart from differences in PD‐L1 expression, there were no significant differences in other clinical and pathological characteristics between these groups.

**TABLE 1:** Baseline Characteristics of HER2‐Mutant patients in first‐line treatment.

Characteristic	Chemotherapy, *n* (%)	Chemotherapy + ICIs, *n* (%)	Chemotherapy + bevacizumab, *n* (%)	Pyrotinib, *n* (%)	*p*‐value
Patients	38	21	20	10	
Mean age, years	57.6 (27–78)	58.3 (38–74)	56.4 (35–73)	56.3 (42–71)	
Gender					
Male	24 (63.2)	12 (57.1)	13 (65.0)	4 (40.0)	0.5541
Female	14 (36.8)	9 (42.9)	7 (35.0)	6 (60.0)	
Smoking status					
Current/former smoker	10 (26.3)	10 (47.6)	8 (40.0)	3 (30.0)	0.3817
Never smoker	28 (73.7)	11 (52.4)	12 (60.0)	7 (70.0)	
PD‐L1 status					
Positive	2 (5.3)	9 (42.9)	2 (10.0)	1 (10.0)	0.0064
Negative	17 (44.7)	7 (33.3)	11 (55.0)	3 (30.0)	
Unknown	19 (50.0)	5 (23.8)	7 (35.0)	6 (60.0)	
Clinical stage					
IIIB/IIIC	3 (7.9)	0	1 (5.0)	0	0.4763
IV	35 (92.1)	21 (100)	19 (95.0)	10 (100)	
Number of metastatic sites					
0	4 (10.5)	2 (9.5)	3 (15.0)	0	0.3196
1	5 (13.2)	4 (19.0)	0	0	
2	8 (21.0)	8 (38.2)	5 (25.0)	4 (40.0)	
3	21 (55.3)	7 (33.3)	12 (60.0)	6 (60.0)	
Brain metastases					
No	34 (89.5)	18 (85.7)	13 (65.0)	6 (60.0)	0.0502
Yes	4 (10.5)	3 (14.3)	7 (35.0)	4 (40.0)	
Pleural metastasis					
No	25 (65.8)	13 (61.9)	13 (65.0)	9 (90.0)	0.4383
Yes	13 (34.2)	8 (38.1)	7 (35.0)	1 (10.0)	
Toxicity graded ≥2					
Yes	11 (28.9)	10 (47.6)	5 (25.0)	5 (50.0)	0.2661
No	27 (71.1)	11 (52.4)	15 (75.0)	5 (50.0)	

**FIGURE 2:**
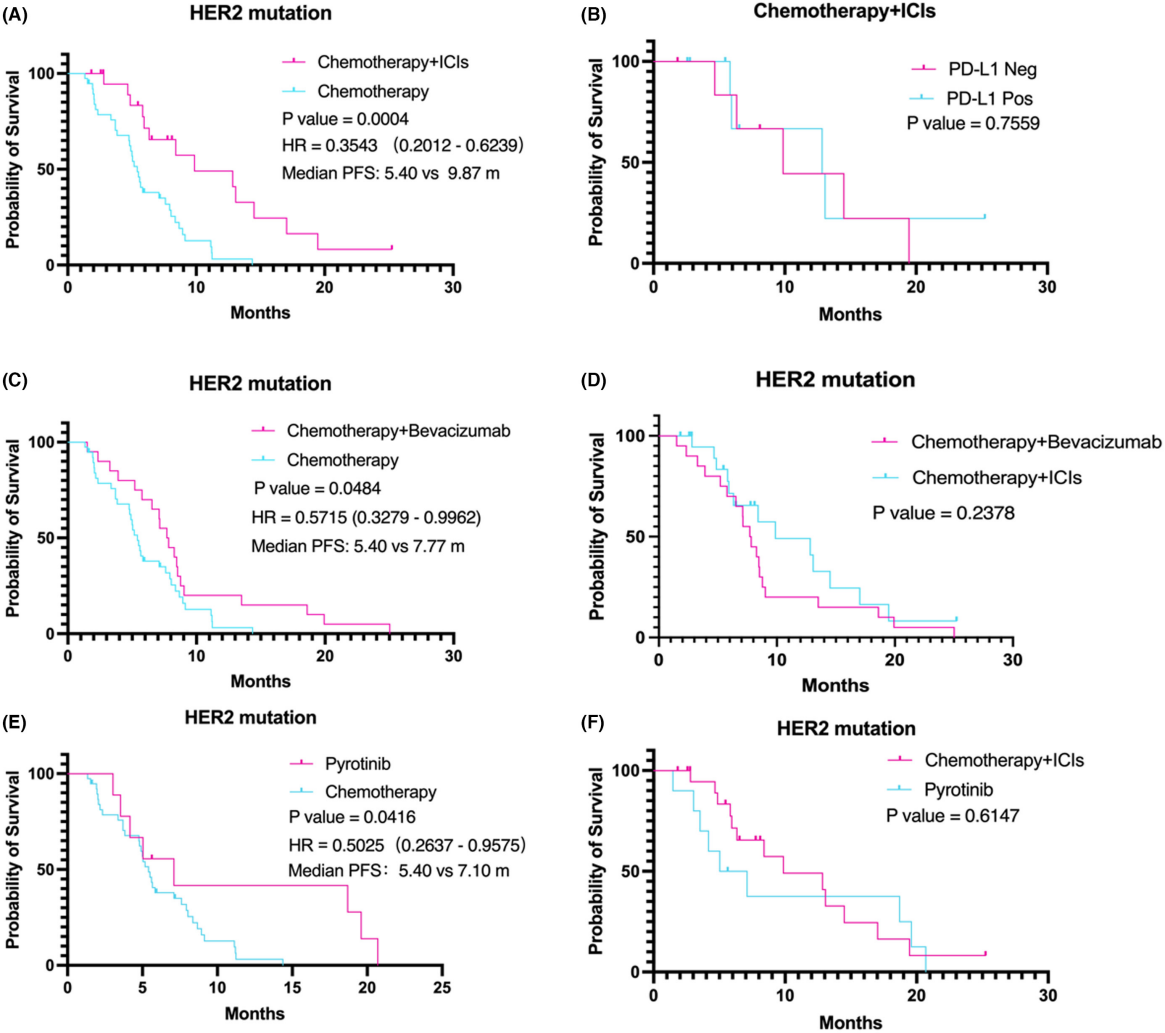
Kaplan–Meier survival curves of PFS in HER2 mutation. (A) Chemotherapy + ICIs versus chemotherapy; (B) PD‐L1 Negative versus PD‐L1 Positive; (C) chemotherapy + bevacizumab versus chemotherapy; (D) chemotherapy + bevacizumab versus chemotherapy + ICIs; (E) pyrotinib versus chemotherapy; (F) pyrotinib versus chemotherapy + ICIs.

We further investigated which first‐line treatment approach confers a survival advantage in patients with HER2‐mutant. The chemotherapy + ICI group demonstrated significantly higher PFS compared to the chemotherapy group (median PFS: 9.87 months vs. 5.40 months, HR 0.3543 (95% CI, 0.2012–0.6239), *p* = 0.0004) (Figure 2A). However, there was no significant difference in PFS between the PD‐L1‐positive and PD‐L1‐negative populations in the chemotherapy + ICI group (Figure 2B). Besides that, a higher PFS was also observed in the chemotherapy + bevacizumab group compared to the chemotherapy group (median PFS: 7.77 months vs. 5.40 months, HR 0.5715 (95% CI, 0.3279–0.9962), *p* = 0.0484) (Figure 2C). However, chemotherapy + bevacizumab group did not demonstrate higher survival benefits compared to chemotherapy + ICI group (Figure 2D). Similarly, compared to chemotherapy, the PFS in the pyrotinib group was significantly better (median PFS: 7.10 months vs. 5.40 months, HR 0.5025 (95% CI, 0.2637–0.9575), *p* = 0.0416) (Figure 2E). The pyrotinib group did not demonstrate an advantage compared to the chemotherapy + ICI group (Figure 2F).

### 3.2 The pyrotinib cohort

A total of 30 patients were identified who met the study inclusion criteria (Figure 1). The median age of the patients was 56.3 years (range, 27–78 years). At baseline, 29 (96.7%) of the 30 patients had stage IV disease. In first‐line treatment, 19 (63.3%) patients had received Chemotherapy, 7 (23.4%) patients had received Chemotherapy + bevacizumab, and 4 (13.3%) patients had received Chemotherapy + ICIs. Clinicopathological characteristics of HER2‐Mutant patients with pyrotinib can be found in Table 2. There were no significant differences in other clinical and pathological characteristics between these groups.

**TABLE 2:** Clinical and pathological characteristics of HER2‐mutant patients treated with pyrotinib after first‐line treatment progression in this study.

Characteristic	All, *n* (%)	First‐line PFS ≤6 months	First‐line PFS >6 months	*p*‐value
Patients	30	17	13	
Mean age, years	56.3 (27–78)	55.8 (27–78)	57 (35–72)	
Gender				
Male	20 (66.7%)	12	8	0.7055
Female	10 (33.3%)	5	5	
Smoking status				
Current/former smoker	6 (20.0%)	2	4	0.3598
Never smoker	24 (80.0%)	15	9	
Clinical stage				
IIIC	1 (3.3%)	1	0	>0.9999
IV	29 (96.7%)	16	13	
Number of metastatic sites				
0–1	10 (16.7%)	7	3	0.4404
2–3	20 (16.7%)	10	10	
Brain metastases				
No	23 (72.6%)	15	8	0.1897
Yes	7 (27.4%)	2	5	
First‐line treatment				
Chemotherapy	19 (63.3%)	13	6	0.1973
Chemotherapy + bevacizumab	7 (23.4%)	3	4	
Chemotherapy + ICIs	4 (13.3%)	1	3	
Pyrotinib treatment line				
2	22 (73.3%)	14	8	0.2420
≥3	8 (26.7%)	3	5	
Best response				
PR	9 (30.0%)	8	1	0.0479
SD	17 (56.7%)	8	9	
PD	4 (13.3%)	1	3	

**FIGURE 3:**
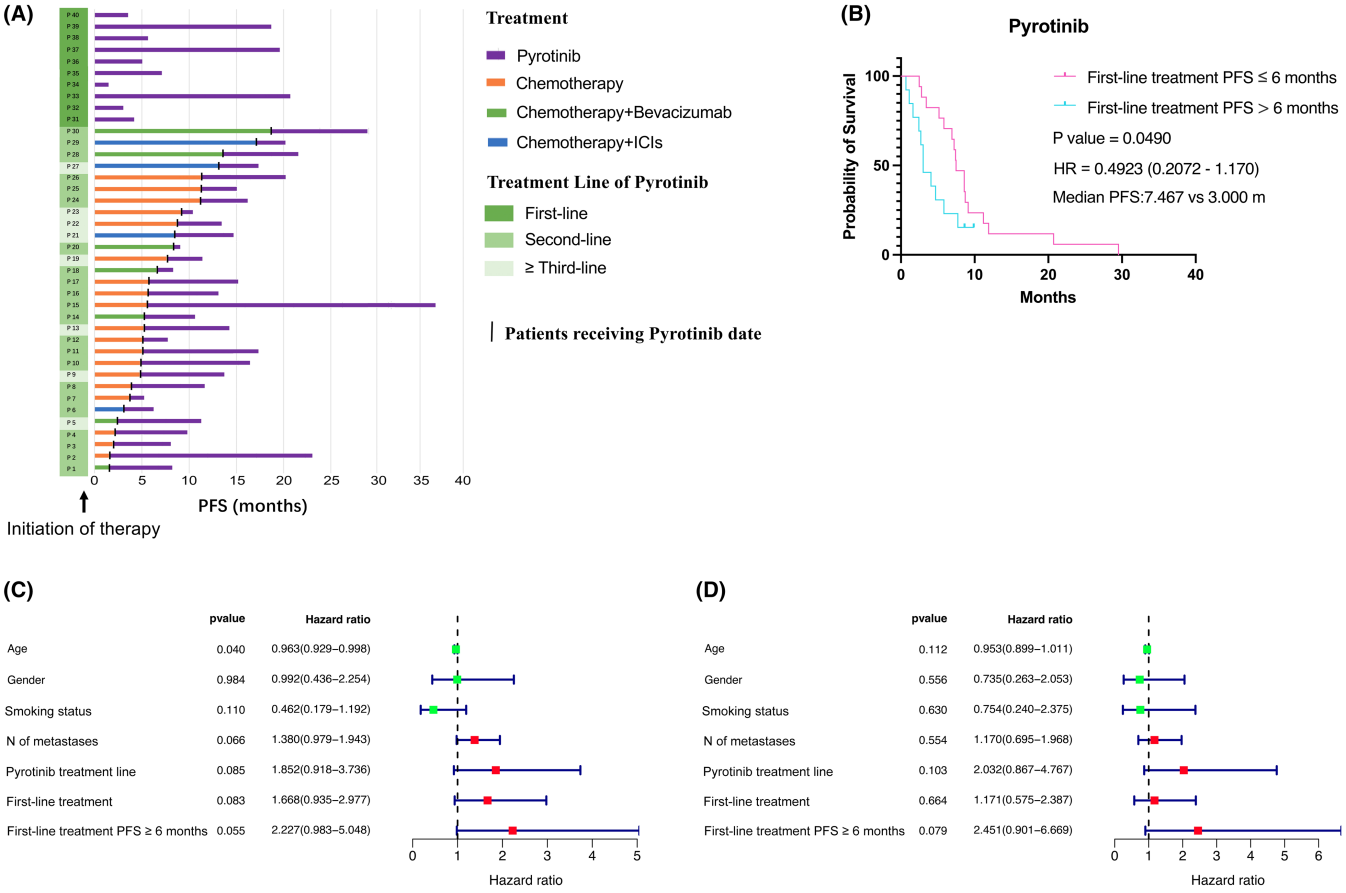
HER2‐mutant patients treated with pyrotinib after first‐line treatment progression. (A) Survival benefits of Pyrotinib based on treatment data and line of treatment. (B) Kaplan–Meier survival curves of PFS based on first‐line treatment PFS; (C) univariate analysis; (D) multivariate analysis.

Subsequently, we further analyzed whether the efficacy of first‐line treatment would impact the subsequent effectiveness of pyrotinib. Exceeding 6 months of PFS with first‐line treatment is considered indicative of acquired resistance to immunotherapy.^29^ We categorized the first‐line treatment PFS within our pyrotinib cohort to determine whether it exceeded the 6‐month threshold (Figure 3A). We conducted a survival analysis on HER2‐Mutant patients receiving pyrotinib and observed that the first‐line treatment PFS of less than 6 months showed a statistical significance (median PFS: 7.467 vs. 3.000, HR 0.4923 (95% CI, 0.2072–1.170), *p* = 0.0490) (Figure 3B). In univariate analysis, the variables influencing NSCLC survival included age (*p* = 0.04) (Figure 3C). We observed that the first‐line treatment PFS of less than 6 months showed a borderline statistical significance in univariate analysis and multivariate analysis (*p* = 0.055 and *p* = 0.079) (Figure 3C,D). In summary, our study findings are similar to previous prospective clinical researc.^25^, ^26^ We have further validated the efficacy of pyrotinib in advanced LUAD with HER2 mutations in the real world. Moreover, we found that patients with poorer first‐line treatment outcomes may benefit from pyrotinib. Further expansion of the sample size is needed to provide more evidence.

### 3.3 Public databases cohort

**FIGURE 4:**
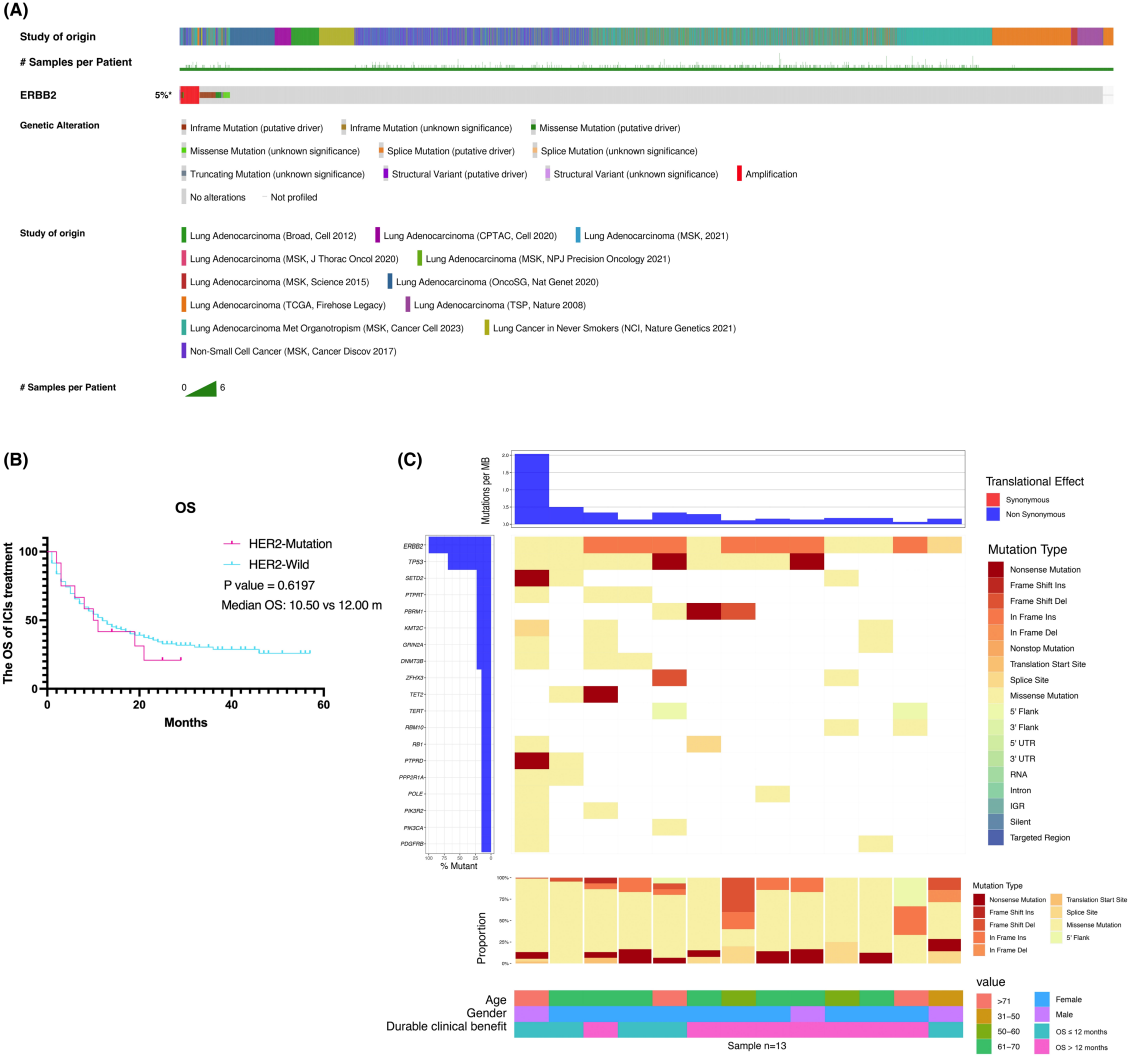
Summary of genomic landscape and clinical features in HER2‐Mutant NSCLC with immunotherapy. (A) Mutation frequency; (B) Kaplan–Meier survival curves of OS; (C) OncoPrint that top 20 gene alterations.

In this study, we included 6290 patients with LUAD, revealing a 5% frequency of HER2 alterations (Figure 4A). Among them, HER‐2 mutations were observed in 209 (3.32%) cases, consistent with previously reported results. Then, Our further investigation revealed that in 350 NSCLC patients receiving immunotherapy, HER2 mutations did not show a trend indicating a survival disadvantage (*p* > 0.5) (Figure 4B). Considering our previous results, which suggest a trend of immunotherapy superiority over other treatments, we anticipate discovering an appropriate biomarker for screening patients eligible for immunotherapy. Therefore, we generated the mutation profile of these 13 HER2‐mutant patients who received immunotherapy. The top gene alterations in HER2‐mutant NSCLC including TP53, SETD2, PTPRT, PBRM1, KMT2C, GRIN2A, DNMT3B, ZFHX3, TET2, TERT, RBM10, RB1, PTPRD, PPP2R1A, POLE, PIK3R2, PIK3CA, PDGFRB (Figure 4C).

## 4 DISCUSSION

This study has two primary objectives: (1) to assess the real‐world safety and effectiveness of first‐line treatment with chemotherapy + ICIs, chemotherapy + bevacizumab, and pyrotinib in advanced LUAD patients with HER2‐mutant; (2) to evaluate the real‐world effectiveness of pyrotinib in advanced LUAD patients with HER2‐mutant. Notably, compared to earlier retrospective studies (Table 3), our study is one of the few studies to analyze the first‐line clinical outcomes of chemotherapy + ICIs, chemotherapy + bevacizumab, and pyrotinib for advanced LUAD with HER2‐Mutant.

**TABLE 3:** Reported retrospective studies on the efficacy of first‐line treatment for advanced NSCLC with HER2‐Mutant.

Author	Published year	Sample size	Treatment group	Control group	Median PFS (Months)	Significant	Reference
Juliana Eng	2016	38	Chemotherapy	NA	7.5	NA	[30]
J Mazières	2016	93	Chemotherapy	NA	6.0	NA	[17]
Zhengbo Song	2016	14	Chemotherapy	NA	4.6	NA	[31]
Yan Wang	2018	25	Chemotherapy	NA	5.1	NA	[15]
Jean‐Bernard Auliac	2019	20	Chemotherapy	NA	6.7	NA	[20]
J Mazieres	2019	29	ICIs alone	NA	2.5	NA	[32]
Florian Guisier	2020	23	ICIs alone	NA	2.2	NA	[33]
Fei Xu	2020	75	Chemotherapy	HER2‐TKIs	5.5	*p* < 0.05	[8]
Jiebai Zhou	2020	33	Chemotherapy	HER2‐TKIs	5.9	*p* > 0.05	[19]
Kaiyan Chen	2021	21	ICIs alone	NA	1.9	NA	[34]
Shuo Yang	2021	82	Chemotherapy	NA	5.8	NA	[35]
Felix C Saalfeld	2021	27	ICIs/ ICIs + Chemotherapy	NA	6.0	NA	[16]
Guangjian Yang	2022	46	Chemotherapy + ICIs	Chemotherapy	5.2	*p* > 0.05	[21]
		81	Chemotherapy + angiogenesis inhibitors	Chemotherapy	5.63	*p* < 0.05	
Xiangling Chu	2022	9	Chemotherapy + ICIs	NA	9.1	NA	[36]
Jiayan Chen	2023	36	Chemotherapy + ICIs	Anti‐ERBB2‐TKI	7.8	*p* < 0.05	[37]

Our study provides comprehensive real‐world evidence on the clinical effectiveness of different treatments for advanced LUAD with HER2 mutations in a first‐line treatment. the median PFS values of the first‐line treatments were 5.40 months for chemotherapy, 9.87 months for chemotherapy + ICIs, 7.77 months for chemotherapy + bevacizumab, and 7.10 months for pyrotinib, respectively. And our research produced some interesting findings. We initially observed that these treatment modalities are all superior to chemotherapy, with chemotherapy + ICIs demonstrating the highest median PFS.^8^, ^15^, ^16^, ^17^, ^20^, ^21^, ^30^, ^32^, ^33^, ^34^, ^35^ PD‐L1 expression was also challenging to identify patients that were suitable for chemotherapy + ICIs. In summary, our real‐world study initially provides preliminary evidence that chemotherapy combined with ICIs demonstrates more favorable efficacy and safety than chemotherapy.

Besides that, pyrotinib has been confirmed to demonstrate significant antitumor activity and reliable safety in solid tumors with HER2 alterations. Therefore, we further collected real‐world evidence on pyrotinib in the first‐line cohort. We hypothesized that patients with poorer first‐line treatment outcomes may benefit from subsequent pyrotinib treatment. Further expansion of the sample size is essential to offer more substantial evidence. This study still has some limitations. Firstly, due to its retrospective nature and considerable heterogeneity in treatment regimens, inherent biases are unavoidable. Secondly, HER2 testing for some patients relied on information from external institutions. Additionally, clinical indicators such as PD‐L1 expression in the majority of patients in this study were unknown, lacking detection information. Finally, we lack an analysis of HER2 mutation subtypes, preventing a comprehensive comparison of clinical benefits among different HER2 mutation subtypes.

In conclusion, our study emphasizes the promising feasibility of chemotherapy + ICIs, chemotherapy + bevacizumab, and pyrotinib as a first‐line treatment option in standard clinical practice for advanced LUAD patients with HER2‐Mutant. No significant distinctions were observed among these three treatment strategies. Efforts should be made to identify efficacy biomarkers that can aid in the selection of suitable therapeutic modalities. Moreover, pyrotinib provided antitumor efficacy in HER2‐Mutant patients with LUAD. And patients with poorer first‐line treatment outcomes may benefit from subsequent pyrotinib treatment. Thus, our results still warrant further larger randomized clinical trials for confirmation.

## AUTHOR CONTRIBUTIONS

**Li Wang:** Data curation (equal); investigation (equal); methodology (equal); software (equal); writing – original draft (equal); writing – review and editing (equal). **Yueran Wu:** Formal analysis (equal); methodology (equal); writing – review and editing (equal). **Zhixuan Ren:** Methodology (equal); software (equal); validation (equal); writing – original draft (equal). **Xiangling Chu:** Data curation (equal); formal analysis (equal); software (equal); writing – review and editing (equal). **Jianing Chen:** Formal analysis (equal); methodology (equal); writing – original draft (equal). **Li Liu:** Methodology (equal); validation (equal); visualization (equal). **Jing Zhao:** Methodology (equal); validation (equal). **Xin Yu:** Software (equal); writing – review and editing (equal). **Mengqing Xie:** Software (equal); validation (equal). **Chunxia Su:** Conceptualization (equal); funding acquisition (equal); project administration (equal); supervision (equal); writing – review and editing (equal).

## FUNDING INFORMATION

This study was supported by National Natural Science Foundation of China (grant numbers: 82072568, 82373320), Shanghai Shenkang development research physician project (grant number: SHDC2022CRD048), and National Key R&D Program of China (grant numbers: 2023YFC2508605).

## CONFLICT OF INTEREST STATEMENT

All authors declare no conflict of interest.

## ETHICS STATEMENT

This single‐center retrospective study was supervised in compliance with the regulations outlined in the Declaration of Helsinki and sanctioned by the Ethics Committee of Shanghai Pulmonary Hospital (Approval number: L21‐304). The hospital ethics committee has agreed to exempt informed consent for retrospective studies.

## CONSENT

Not applicable.

## Data Availability

The datasets used and/or analyzed during the current study are available from the corresponding author upon reasonable request.
